# Prevalence of undetectable and suppressed viral load in HIV-infected pregnant women initiating Option B+ in Uganda: an observational study nested within a randomized controlled trial

**DOI:** 10.1186/s12879-021-06608-4

**Published:** 2021-09-04

**Authors:** Grace Gabagaya, Gordon Rukundo, Alexander Amone, Priscilla Wavamunno, Joyce Namale-Matovu, Irene Lubega, Clemensia Nakabiito, Zikulah Namukwaya, Monica Nolan, Samuel S. Malamba, Rachel King, Jaco Homsy, Mary Glenn Fowler, Philippa Musoke

**Affiliations:** 1 Makerere University - Johns Hopkins University Research Collaboration, P.O. Box 23491, Kampala, Uganda; 2 Uganda Virus Research Institute, Entebbe, Uganda; 3 Institute for Global Health Sciences, University of California, San Francisco, USA; 4 Department of Pathology, Johns Hopkins University, School of Medicine, Baltimore, USA; 5 Department of Paediatrics & Child Health, School of Medicine, College of Health Sciences, Makerere University, Kampala, Uganda

**Keywords:** HIV, Pregnancy, Viral load, Viral or virological suppression, Uganda, HIV transmission, Option B+, Antiretroviral therapy

## Abstract

**Background:**

Viral load (VL) testing is key in monitoring adherence to antiretroviral therapy (ART) and documenting HIV treatment response. As per HIV treatment guidelines in Uganda, the first VL test is recommended 6 months after initiation of ART. Undetectable VL (uVL) at ART initiation may be helpful in detecting elite controllers in the absence of previous ART use. We investigated viral suppression at ART initiation among a cohort of HIV-positive pregnant women enrolled in the Friends for Life Circles (FLC) for Option B+ randomized controlled trial (RCT).

**Methods:**

Pregnant women ≥ 18 years of age testing positive for HIV at their first antenatal care visit and starting on ART Option B+ as per the National PMTCT Program guidelines were enrolled into the FLC for Option B+ RCT in urban Kampala and rural Mityana districts of Uganda. Each participant had whole blood samples collected at enrolment to assess baseline VL. Plasma HIV-1 RNA was quantified using COBAS Ampliprep /COBAS Taqman. Baseline VL below 400 RNA copies/ml was considered as viral suppression while baseline VL below 20 RNA copies/ml was considered uVL.

**Results:**

The mean duration from the date of ART initiation to time of sample collection for baseline VL assessment was 4.4 days (SD 3.6). Of the 532 HIV-positive pregnant women enrolled in the FLC for Option B+ study and newly starting Option B+ without a self-reported history of prior ART use, 29 (5.5%) had uVL and 113 (21.4%) had suppressed VL at baseline. There was no association between participants’ age, gravidity, marital status, mean monthly income, educational level, disclosure of HIV status to partner, and uVL or viral suppression at baseline. However, non-disclosure of HIV status to any other person was associated with decreased odds of viral suppression at baseline (OR 0.640; 0.416–0.982).

**Conclusion:**

Twenty-one percent of HIV-positive Ugandan pregnant women initiating ART (Option B+) showed virological suppression at baseline and were presumed to be “elite controllers” or to have misreported being ART-naive. Further studies are needed to better understand the biologic mechanisms of elite controllers among pregnant women as well as to differentiate elite controllers from concealed ART use.

*Trial Registration* The trial was registered as NCT02515370 (04/08/2015) on Clinicaltrials.gov.

## Background

Viral load testing is the gold standard for HIV treatment monitoring and is recommended as the preferred approach to diagnose and confirm treatment failure [1, 2]. The WHO recommends that routine VL testing should be done at 6 and 12 months after initiation of ART for adult HIV patients and every 12 months thereafter if the patient is stable on ART [1].

Given that VL assessment is not done routinely at ART initiation, ART-naïve HIV-infected individuals with suppressed or undetectable VL (uVL) are not identified. Detecting suppressed/undetectable VL in HIV-infected individuals newly initiating ART may be useful in identifying “elite controllers” or unreported ART use. In this paper, we analysed the prevalence of virological suppression at ART initiation among a cohort of HIV-positive pregnant women enrolled in the Friends for Life Circles (FLC) for Option B+ randomized controlled trial (RCT).

## Methods

### Study design and population

The FLC for Option B+ study was an open-label RCT with two parallel intervention and control arms. This RCT was undertaken in South Central Uganda, at Mulago National Referral Hospital and Kampala Capital City Authority (KCCA) health units in Kampala (urban) district as well as at Mityana District hospital and six surrounding health centers in Mityana (rural) district. The RCT goal was to test an enhanced peer group support system called “Friends for Life Circles” (FLCs) to help HIV-infected pregnant women initiating PMTCT Option B+ during the antenatal period adhere to lifelong ART and postpartum care visits [3]. Eligible participants were HIV-positive pregnant women ≥ 18 years of age, newly initiating ART Option B+, who agreed to come to the study clinic for scheduled appointments and to be home visited as needed to ensure follow up [3]. In this RCT, ART-naïve women were defined as those who started on ART on or shortly (≤ 14 days) before their enrolment into the FLC Option B+ trial. Additional inclusion criteria included residing within a 20 km radius around the study clinic and not planning to move out of the catchment area within the next 2 years [3]. Exclusion criteria included any medical, social or other circumstancse that might prevent a mother from adhering to the study protocol [3]. At trial commencement, the study relied on documented pregnancy and positive HIV test from the health units which resulted in enrolment of some participants that were neither pregnant nor HIV positive. The protocol was subsequently amended so that confirmatory testing for pregnancy and HIV positivity could be done by the study prior to enrolment.

### Data collection procedures

Pregnant women testing HIV-positive and started on ART on the same day as part of the National PMTCT Program were screened for the RCT. Eligible women were enrolled and randomized into the RCT within 14 days of ART initiation. At enrolment into the RCT, each participant had whole blood samples collected for assessment of baseline plasma HIV-RNA as well as standardized questionnaires administered to capture data on socio-demographic characteristics, adherence to ARVs and HIV status disclosure.

### Blood sample collection and HIV testing

Whole blood collected in sterile EDTA tubes was stored at room temperature. Plasma was then separated from whole blood by centrifugation within 24 h of sample collection after which plasma was transferred to sterile cryovials. In Kampala, processing and storage was done at the VL testing Laboratory while in Mityana, plasma was stored frozen at – 20 °C in the Mityana Hospital Laboratory and transported to Kampala for storage and VL testing every 1–2 weeks on frozen icepacks in temperature-controlled cool boxes. Plasma VL testing was conducted in batches at the Infectious Disease Institute (IDI) Core Lab in Mulago Hospital using COBAS Ampliprep /COBAS Taqman HIV-1 test whose lower limit of detection was 20 copies/ml. Participants in whom no viral copies were detected had repeat rapid HIV antibody testing done to confirm HIV diagnosis and if found negative, had HIV DNA PCR testing done. Undetectable viral load in this study was defined as VL count < 20 copies/ml while virological suppression was defined as VL count < 400 copies/ml.

### Statistical analysis

STATA software (StataCorp LP, 4905 Lakeway Drive, College Station, TX, USA) was used for all statistical analyses. All collected data on socio-demographic variables were considered for inclusion in the univariate logistic regression analysis and variables with a significance level of p < 0.1 were considered for inclusion in the full multivariate model. Using a backward elimination method, only variables with a significance level of p < 0.05 were kept in the final model. Odds ratios were used to measure the magnitude of the effect and a Wald test was used to evaluate the statistical significance of each coefficient in the model assessing the relationship between socio-demographic characteristics and the different binary viral load outcome groups [(0 = detectable VL, 1 = undetectable VL) or (0 = unsuppressed VL, 1 = suppressed VL)]. The likelihood ratio test (LHRT) was used to compare the goodness of fit for two nested models.

## Results

A total of 540 HIV-positive pregnant women participants were enrolled into the FLC for Option B+ RCT between May 2016 and September 2017. Of these, eight participants were subsequently terminated due to inappropriate enrolment and are excluded from the data analyses. Three of these did not disclose information on ineligibility status during screening while five of these were found to be neither pregnant nor HIV-positive upon confirmatory testing.

The mean duration from date of ART initiation to time of sample collection for baseline VL assessment was 4.4 days (SD 3.6) and the median was 4 days (range 0–30 days, IQR 2–6 days). Table 1 shows the univariate analysis of the proportions of the 532 eligible study participants with baseline suppressed (< 400 copies/ml) and undetectable (< 20 copies/ml) HIV VL by socio-demographics characteristics.

**Table 1:** Proportions of pregnant women with baseline suppressed and undetectable HIV viral load by socio-demographic characteristics

Characteristics	N = 532	Column %	Suppressed VL (< 400 copies/ml)		p-value	Undetectablevl (< 20 copies/ml)		p-value
			n	Row%		n	Row%	
Site								
Urban-Kampala	395	74.2	87	22.0		19	4.8	
Rural-Mityana	137	25.8	26	19.0	0.452	10	7.3	0.269
Age in years								
18–24	256	48.1	57	22.3		13	5.1	
25–34	238	44.8	50	21.0		14	5.9	
35–44	38	7.1	6	15.8	0.314	2	5.3	0.924
Marital status								
Married/co-habiting	424	79.7	89	21.0		25	5.9	
Never married	85	16.0	23	27.1		4	4.7	
Separated/divorced/widowed	23	4.3	1	4.4	0.483	0	0.0	0.454
Educational level								
University/College/Tertiary	24	4.5	3	12.5		0	0.0	
Secondary^b^	257	48.3	57	22.2		15	5.8	
Primary^b^	228	42.9	48	21.1		13	5.7	
No formal education	23	4.3	5	21.7	0.422	1	4.4	0.673
Religion								
Catholic	200	37.6	46	23.0		10	5.0	
Protestant	123	23.1	26	21.1		10	8.1	
Moslem	109	20.5	20	18.4		6	5.5	
SDA/Pentecostal/others	100	18.8	21	21.0	0.076	3	3.0	0.398
Tribe								
Non-Ganda	235	44.2	55	23.4		13	5.5	
Ganda	297	55.8	58	19.5	0.775	16	5.4	0.942
Gravidity								
1	100	18.8	23	23.0		2	2.0	
2	127	23.9	31	24.4		11	8.7	
3	140	26.3	29	20.7		9	6.4	
4 +	165	31.0	30	18.2	0.353	7	4.2	0.134
Disclosure to partner								
Yes	213	40.0	52	24.4		13	6.1	
No	302	56.8	58	19.2		16	5.3	
No partner	17	3.2	3	17.7	0.424	0	0.0	0.557
Disclosure to other								
Yes	201	37.8	52	25.9		15	7.5	
No	331	62.2	61	18.4	0.066	14	4.2	0.111
Having any source of income^c^								
No	309	58.2	67	21.7		19	6.2	
Yes	222	41.8	45	20.3	0.694	9	4.1	0.287
Total	532	100.0	113	21.3		29	5.5	

^a^All 532 study participants included in the analysis were started on ART 30 days or less before being bled for VL count. The median number of days from ART initiation to enrolment was 4 days (Range 0–30 days, IQR: 2–6 days); the mean was 4.4 days (SD: 3.6). The 8 individuals who were inappropriately enrolled in the study are excluded from this analysis

^b^Partial or completed level of education

^c^One study participant had no information on source of income

Participants’ median age was 25 years; 137 (25.8%) were from rural Mityana and 395 (74.2%) from urban Kampala. Twenty-nine (5.5%) of 532 enrolled women had baseline uVL (< 20 copies/ml) and 84 (15.8%) had baseline viral suppression (20–399 copies/ml), all with no self-reported prior ART use. Therefore, the prevalence of undetectable VL was 5.5% and the proportion of patients with suppressed VL (< 400 copies/ml) was 21.2%. The median VL counts and frequencies of the different VL categories by RCT site are summarized in Table 2.

**Table 2:** Baseline VL and distribution of pregnant women across VL ranges in Kampala and Mityana districts

Baseline VL counts and distribution	Enrolled women in Kampala (urban)		Enrolled women in Mityana (rural)		All enrolled women (urban & rural)	
Median baseline VL (cpml, IQR)—all cpml ranges	3695 (588–18,366)		7585 (1001–33,081)		4480.5 (638–21,420.5)	
Distribution of women across different VL ranges (cpml)	N = 395	Column (%)	N = 137	Column (%)	N = 532	Column (%)
< 20	19	4.8	10	7.3	29	5.5
20–399	68	17.2	16	11.6	84	15.8
400–999	34	8.6	8	5.8	42	7.9
≥ 1000	274	69.4	103	75.2	377	70.9

The median age in years for participants with and without uVL at baseline was 25 (IQR: 21, 30) and 25 (IQR: 22, 30) respectively. The multivariate analysis (Table 3) shows that disclosure of HIV status to any person other than a partner was the only factor associated with baseline VL suppression (VL < 400 copies/ml); there was no association between gravidity, marital status, religion, tribe, mean monthly income, educational level attained, disclosure of HIV status to partner and uVL (VL < 20 copies/ml) or VL suppression (VL < 400 copies/ml) at baseline as illustrated in Table 3. The adjusted odds ratio for VL suppression (< 400 copies/ml) in those who did not disclose compared to those who disclosed to any person other than their partner was 0.640 (95% CI 0.416–0.982, p = 0.041), after adjusting for marital status. (Table 3).

**Table 3:** Multivariate analysis of factors associated with baseline VL suppression in FLC for Option B+ RCT participants

Characteristics	Unadjusted OR (95% CI)	p-value	Adjusted OR^a^ (95% CI)	p-value
Site				
Urban-Kampala	1.000			
Rural-Mityana	0.829 (0.509–1.352)	0.453		
Age				
18–24 years	1.000			
25–34 years	0.929 (0.605–1.425)	0.735		
35 + years	0.655 (0.261–1.643)	0.367		
Marital status				
Married/co-habiting	1.000		1.000	
Never married	1.396 (0.820–2.378)	0.219	1.275 (0.742–2.192)	0.379
Separated/divorced/widowed	0.171 (0.023–1.287)	0.086	0.153 (0.020–1.156)	0.069
Educational level				
University/College/Tertiary	1.000			
Secondary^b^	1.995 (0.574–6.928)	0.277		
Primary^b^	1.867 (0.534–6.521)	0.328		
No education	1.944 (0.407–9.287)	0.405		
Religion				
Catholic	1.000			
Protestant	0.897 (0.521–1.546)	0.696		
Moslem	0.752 (0.419–1.352)	0.341		
SDA/Pentecostal/others	0.890 (0.497–1.594)	0.695		
Tribe				
Non-Ganda	1.000			
Ganda	0.794 (0.524–1.204)	0.278		
Gravidity				
1	1.000			
2	1.081 (0.583–2.000)	0.804		
3	0.875 (0.471–1.625)	0.672		
4 +	0.744 (0.404–1.371)	0.343		
Disclosure to partner				
Yes	1.000			
No	0.736 (0.482–1.125)	0.156		
No partner	0.663 (0.183–2.399)	0.532		
Disclosure to other^c^				
Yes	1.000		1.000	
No	0.647 (0.425–0.986)	0.043	0.640 (0.416–0.982)	0.041
Any source of income				
No	1.000			
Yes	0.542 (0.224–1.316)	0.176		

^a^Variables that had unadjusted p-value > 0.10 were not included in adjusted model

^b^Partial or completed level of education

^c^We compared disclosure to other using the likelihood ratio test (LHRT) and the goodness of fit of a reduced model that considered only the disclosure to people who are not a woman’s partner(s) with the final model that considered both disclosure to people other than a woman’s partner(s) and marital status variables. We obtained a LHRT of 7.07 and a p-value of 0.0291. This suggests that the two models were not equivalent and that the final model had a better fit to the data

## Discussion

Our results show that 5.5% of HIV-infected pregnant women who initiated ART in the study had uVL at baseline and were potential elite controllers (EC). This is higher than previous findings in Uganda where a prevalence of 1% and 0.26% of HIV controllers (ECs and aviraemic controllers) were reported by Laeyendecker et al. [4] and Kayongo et al. [5] in 2009 and 2018 respectively. However, our findings are consistent with the findings of a 2014 systematic review where the proportion of ECs was 0.15–7.7% and did not necessarily reflect the length of follow-up [6].

ECs are HIV-positive individuals who are able to suppress viral replication to undetectable levels for extended periods of time without ART. This definition of elite controllers thus requires factoring in longitudinal characteristics of VL suppression over a certain period of time [6, 7], which was not documented in our study since it was a baseline analysis. ECs are believed to control viral replication through mechanisms involving host immune responses [6]. Consistent with our findings, previous reports have not documented any association between elite controllers and specific socio-demographic characteristics [7].

We also identified a high proportion of women who were virally suppressed (< 400 copies/ml) at baseline (N = 113, 21.2%). This is in contrast to the HPTN068 study [8] which, in spite of using a higher threshold for viral suppression (< 2000 copies/ml), had only 15.7% of patients virally suppressed in the absence of ART. In our study, women who had not disclosed to an other person than their partner were less likely to be virally suppressed (OR 0.64; 95% CI 0.416–0.982, p = 0.041) than those who had disclosed. It is difficult to interpret this finding without more information but disclosure of HIV remains a challenge in Uganda because of stigma, particularly for women bearing children for fear of the consequences such women might face from their partner or others [9]. It might indicate that some of these women have been infected for some time and did not disclose nor get appropriate care and treatment for fear of discrimination from their social network [10].

It is also possible the women failed to report prior ART use since our findings are based on self-report. Much as we found no association between gravidity and VL suppression, there is a possibility that women with previous pregnancies after 2012 when Uganda rolled out Option B, could have received ART for PMTCT and developed viral suppression. Findings elsewhere have documented under-reporting of ART use by HIV patients [11–13]. In South Africa, blood donors reporting no history of ART use and identified as HIV-positive with undetectable viral load, were presumed ECs. However, when plasma samples of these presumed ECs were later tested for ARVs, undisclosed ART use was discovered in 66.4% of participants [11].

In addition to stigma mentioned above, other possible reasons why women may not report prior ART use are that they may have defaulted on their ART regimen and are thus concerned about being reprimanded. Additionally, participation in a study often implies better care which may prompt women to conceal prior ART use.

Our study had a number of strengths: first, the sample size was relatively large and included both rural and urban Ugandan populations, which increases the generalizability of our results in Uganda. Second, we used a highly sensitive validated assay for measurement of viral load and thus were able to measure VL copies at a very low level of detection. Finally, we were able to conduct HIV DNA PCR testing to confirm HIV status and to guard against including women incorrectly diagnosed as HIV positive based on a falsely positive rapid HIV screening test.

Our study however had some limitations. First, plasma drug levels were not yet available prior to ART initiation and baseline hair collection for measurement of ARV drug concentrations was not done. Baseline hair samples would have been helpful in assessing prolonged ARVs exposure and validation of ART naïve self-reports. Also, baseline VL assessment was done after ART initiation which could raise the question as to whether these women were truly ART-naïve at the time of VL measurement. However, the mean duration from date of ART initiation to time of blood sample collection was 4.4 days (median 4 days, range 0–30 days, IQR 2–6 days) which is very unlikely to have impacted VL measurements, as a period of 5 to 15 weeks has been reported as time to uVL after initiation of ART among HIV-infected pregnant women [14]. We also did not have data on CD4 levels because, since the adoption of Option B+ for PMTCT and the introduction of test and treat HIV policy in Uganda, ART is initiated immediately after confirming HIV infection without the need to measure CD4 levels. Additionally, our multivariate analysis only evaluated suppressed viral load (< 400 copies/ml) but not undetectable viral load because of small numbers in the undetectable category (< 20 copies/ml + TND). Lastly, much as partner and relatives’ HIV and ART status are important determinants of viral suppression, we did not have these data. This is incumbent on the premise that HIV disclosure and stigma are still challenges and many pregnant women do not share this information.

## Conclusions

In the FLC for Options B+ RCT, over 5% of pregnant HIV-positive women initiating ART (Option B+) had undetectable viral load at baseline and were presumed to be either potential “elite controllers” or did not report previous use of ART. No specific maternal socio-demographic factors aside from disclosure of HIV status to person others than their partner was associated with baseline uVL. Further studies are needed to better understand the biological mechanisms of pregnant women who may be elite controllers; as well as the need and feasibility of screening for baseline antiretroviral drug detection among self-reporting ART-naïve pregnant women with uVL, in order to better inform their care and treatment.

## Data Availability

The datasets used and/or analysed during the current study are available from the corresponding author upon reasonable request. The study protocol has been provided under related files.

## Abbreviations

VL: Viral load
ART: Antiretroviral therapy
uVL: Undetectable VL
FLC: Friends for Life Circles
EC: Elite controllers
